# NFE2L2 and ferroptosis resistance in cancer therapy

**DOI:** 10.20517/cdr.2024.123

**Published:** 2024-10-25

**Authors:** Daolin Tang, Rui Kang

**Affiliations:** Department of Surgery, University of Texas Southwestern Medical Center, Dallas, TA 75390, USA.

**Keywords:** Cancer therapy, drug resistance, ferroptosis, NFE2L2, oxidative stress

## Abstract

NFE2-like basic leucine zipper transcription factor 2 (NFE2L2, also known as NRF2), is a key transcription factor in the cellular defense against oxidative stress, playing a crucial role in cancer cell survival and resistance to therapies. This review outlines the current knowledge on the link between NFE2L2 and ferroptosis - a form of regulated cell death characterized by iron-dependent lipid peroxidation - within cancer cells. While NFE2L2 activation can protect normal cells from oxidative damage, its overexpression in cancer cells contributes to drug resistance by upregulating antioxidant defenses and inhibiting ferroptosis. We delve into the molecular pathways of ferroptosis, highlighting the involvement of NFE2L2 and its target genes, such as *NQO1*, *HMOX1*, *FTH1*, *FTL*, *HERC2*, *SLC40A1*, *ABCB6*, *FECH*, *PIR*, *MT1G*, *SLC7A11*, *GCL*, *GSS*, *GSR*, *GPX4*, *AIFM2*, *MGST1*, *ALDH1A1*, *ALDH3A1*, and *G6PD*, in ferroptosis resistance. Understanding the delicate balance between NFE2L2’s protective and deleterious roles could pave the way for novel therapeutic strategies targeting NFE2L2 to enhance the efficacy of ferroptosis inducers in cancer therapy.

## INTRODUCTION

**Figure 1:**
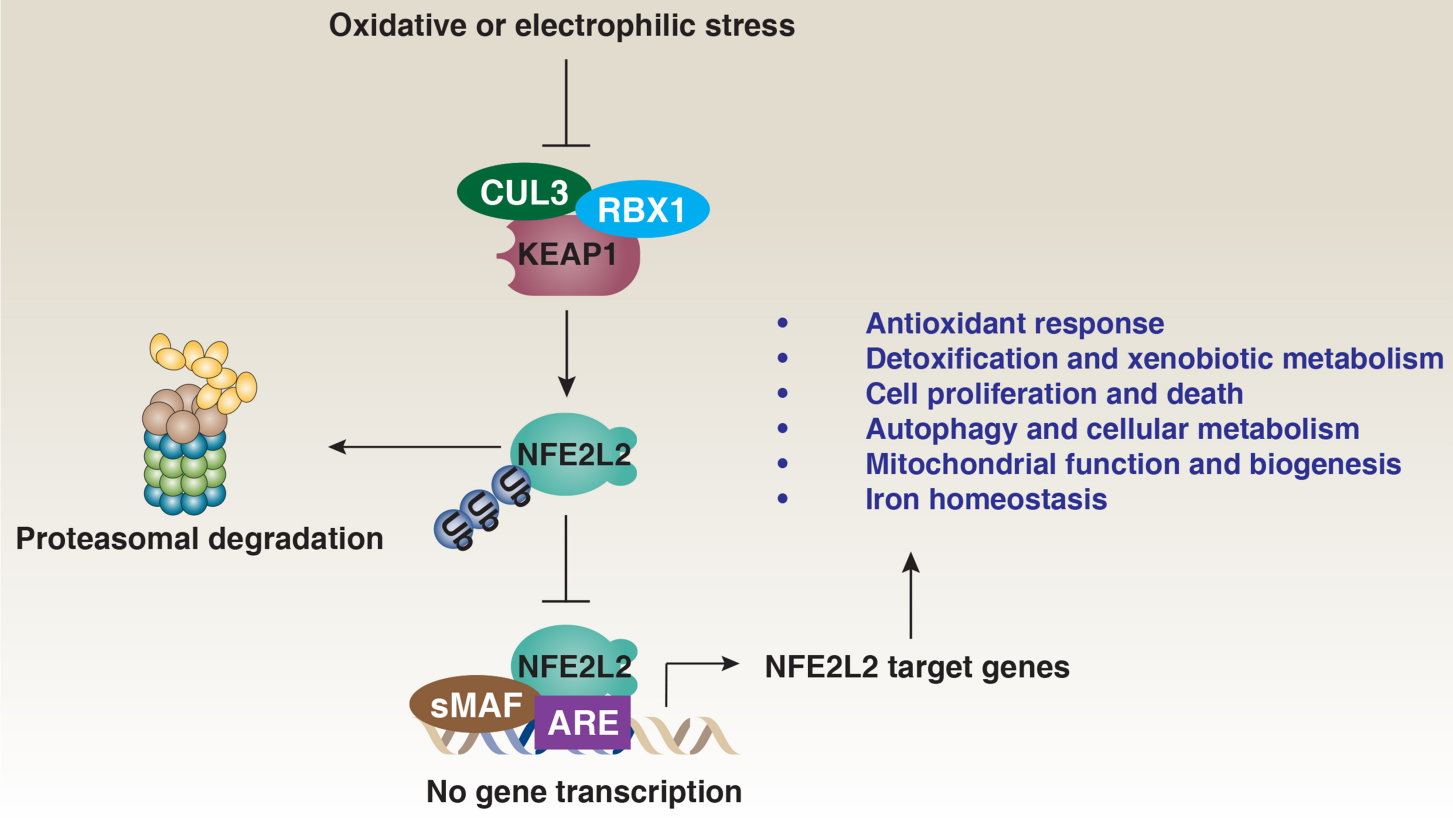
The KEAP1-NFE2L2 signaling pathway. NFE2L2 is primarily regulated by the KEAP1-CUL3-RBX1 E3 ubiquitin ligase complex, which, under normal conditions, promotes the ubiquitination and proteasomal degradation of NFE2L2, keeping its cellular levels low. However, in response to oxidative stress or electrophilic agents, key cysteine residues on KEAP1 are modified, preventing NFE2L2 ubiquitination. As a result, NFE2L2 stabilizes and accumulates in the cytoplasm before translocating to the nucleus. There, it dimerizes with sMAF proteins and binds to AREs in the promoters of target genes. These genes regulate a variety of crucial cellular processes, including antioxidant defense, detoxification, cell proliferation and death, autophagy, metabolism, mitochondrial function, and iron homeostasis. Through these pathways, NFE2L2-mediated gene expression plays an essential role in protecting cells from oxidative damage, maintaining redox balance, and ensuring overall cellular homeostasis. KEAP1: Kelch-like ECH-associated protein 1; NFE2L2: NFE2-like basic leucine zipper transcription factor 2; CUL3: cullin 3; RBX1: ring-box 1; sMAF: small musculoaponeurotic fibrosarcoma; AREs: antioxidant response elements.

NFE2-like basic leucine zipper transcription factor 2 (NFE2L2, also known as NRF2) is a key member of the Cap’n’Collar (CNC) family of basic leucine zipper transcription factors, which share a conserved CNC domain crucial for DNA binding and dimerization. NFE2L2 plays a vital role in cellular defense against oxidative stress^[1]^. Under normal conditions, NFE2L2 levels are tightly controlled and kept low through rapid degradation by the ubiquitin-proteasome system [Figure 1]. Specifically, the E3 ubiquitin ligase Kelch-like ECH-associated protein 1 (KEAP1), part of the cullin 3 (CUL3)-based E3 ubiquitin ligase complex, targets NFE2L2 for degradation by binding to its Neh2 domain, which contains two KEAP1 binding sites (ETGE and DLG)^[2]^. However, in response to oxidative or electrophilic stress, NFE2L2 stability increases, allowing it to accumulate in the nucleus. There, NFE2L2 dimerizes with small musculoaponeurotic fibrosarcoma (sMAF) proteins and binds to antioxidant response elements (AREs) in the promoters of target genes, leading to the expression of a wide range of detoxifying and antioxidant enzymes^[3]^.

NFE2L2 plays a protective role in a wide range of human diseases, including cancer, neurodegenerative disorders, cardiovascular diseases, metabolic syndromes, chronic inflammatory conditions, liver and renal diseases, and infections^[4]^. In cancer, NFE2L2 exhibits a dual function depending on the disease context. In normal cells, it safeguards against oxidative damage by inducing antioxidant and detoxifying enzymes^[5]^. However, in cancer cells, overactivation of NFE2L2 - often due to mutations in KEAP1 or gain-of-function mutations in NFE2L2 - leads to enhanced tumor survival by promoting antioxidant defenses, metabolic reprogramming, and resistance to oxidative stress-induced cell death, such as ferroptosis and apoptosis^[6]^. This constitutive upregulation is common in cancers, including non-small cell lung cancer, pancreatic ductal adenocarcinoma, hepatocellular carcinoma (HCC), and ovarian, bladder, endometrial, and prostate cancers^[7]^. In these malignancies, elevated NFE2L2 activity supports tumor progression, metastasis, and resistance to therapies. As a result, NFE2L2 has become a key target in cancer research, with ongoing efforts to develop activators for conditions requiring enhanced oxidative defense, such as neurodegenerative diseases, and inhibitors to counteract its role in tumor resistance and poor prognosis^[8]^.

Ferroptosis is a form of regulated cell death characterized by the iron-dependent accumulation of lipid peroxides, which leads to oxidative damage and cell death^[9,10]^. Unlike caspase-mediated apoptosis, ferroptosis is primarily driven by the failure of the cell’s antioxidant systems, particularly the glutathione (GSH)-dependent glutathione peroxidase 4 (GPX4) pathway, which normally protects against lipid peroxidation^[11]^. Ferroptosis has emerged as a promising target in cancer therapy, especially for treating tumors that are resistant to other forms of cell death, such as apoptosis^[12,13]^. However, the development of resistance to ferroptosis presents a significant challenge to the efficacy of these therapies.

This review will first provide an overview of the molecular pathways involved in ferroptosis, followed by an in-depth discussion of how NFE2L2 is activated and contributes to drug resistance during ferroptosis-mediated cancer therapy. Understanding these mechanisms may facilitate the development of novel combination therapies designed to enhance the efficacy of ferroptosis inducers and improve anticancer outcomes.

## OVERVIEW OF FERROPTOSIS PATHWAY

Ferroptosis was initially characterized as a targeted form of cell death designed to selectively eliminate cells harboring mutant *RAS* oncogenes^[14]^. The discovery of ferroptosis was driven by the identification of two key compounds, erastin and RSL3, from a large compound library, both of which were found to trigger ferroptosis and inhibit tumor growth in *RAS*-mutant cells^[14,15]^. Early studies highlighted the selective nature of ferroptosis in *RAS*-mutant cells, which generated interest due to its apparent safety for normal cells. However, subsequent research has demonstrated that ferroptotic damage can also occur in normal cells or tissues, implicating ferroptosis in various pathological conditions, including neurodegenerative diseases and ischemia-reperfusion injury^[16,17]^.

Recent findings have further complicated the role of ferroptosis in cancer therapy, suggesting that traditional ferroptosis inducers can not only kill cancer cells but may also impair antitumor immune cells, such as CD8^+^ T cells^[18,19]^, dendritic cells^[20]^, and natural killer cells^[21]^, thereby weakening the overall antitumor immune response. This issue arises because ferroptosis inducers such as erastin and RSL3 target solute carrier family 7 member 11 (SLC7A11) and GPX4 - proteins expressed in both normal and cancer cells. To address this challenge, a novel cell-type-specific ferroptosis inducer, N6F11, was developed to selectively target cancer cells by binding to tripartite motif-containing 25 (TRIM25) and promoting TRIM25-dependent degradation of GPX4^[22]^. Because TRIM25 is primarily expressed in cancer cells and shows low or no expression in immune cells, N6F11 does not adversely affect the immune system^[22,23]^.

**Figure 2:**
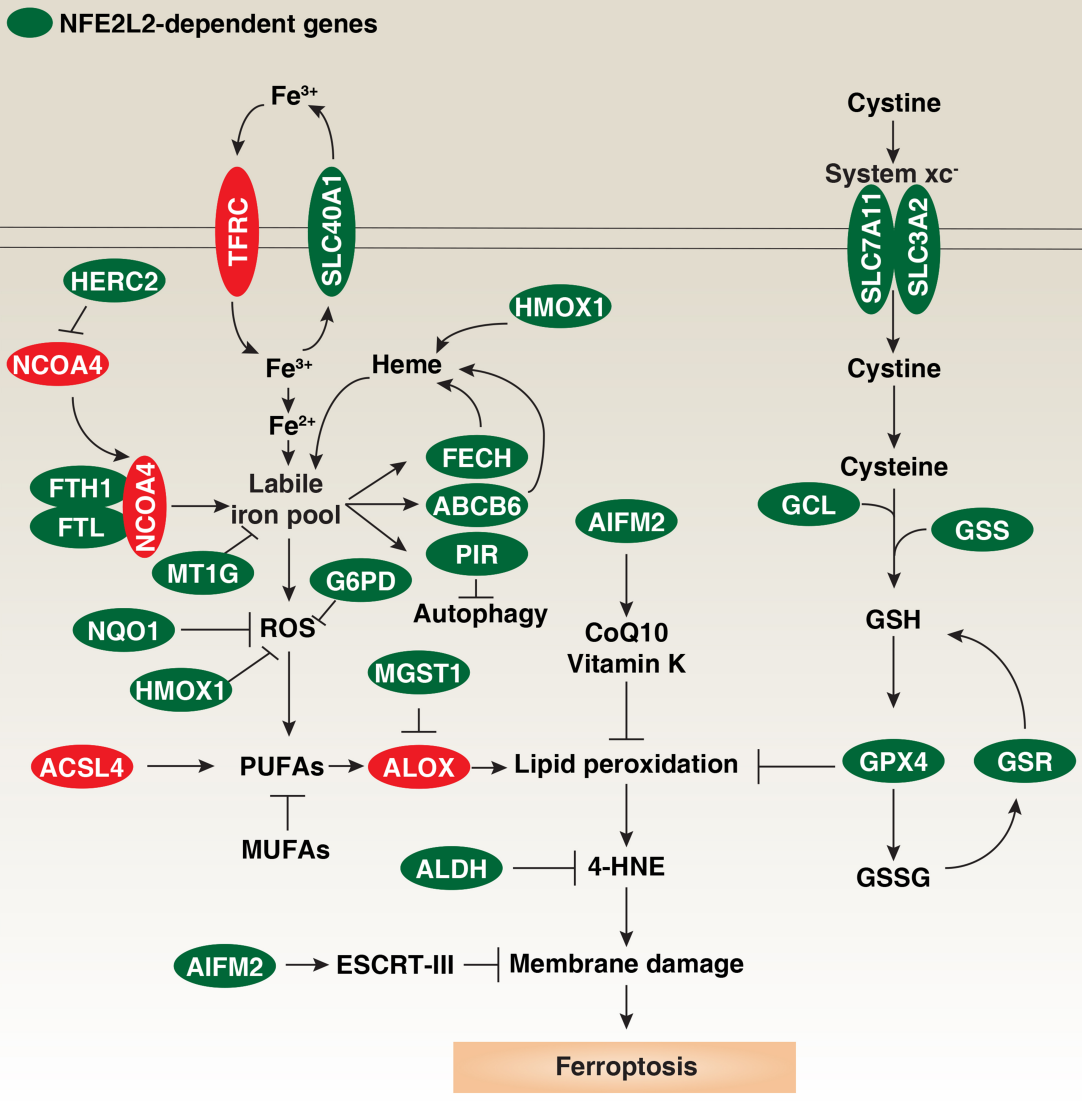
NFE2L2-dependent genes in ferroptosis. Ferroptosis, a form of oxidative cell death, can be triggered by increased iron accumulation or the inhibition of antioxidant systems. Fe^3+^ is taken up by TFRC and exported by SLC40A1. Inside the cell, Fe^3+^ is reduced to Fe^2+^, with excess iron stored in ferritin (composed of FTH1 and FTL) or sequestered by MT1G. Conversely, autophagic degradation of ferritin via NCOA4 can elevate the labile iron pool, a process regulated by HERC2-mediated degradation of NCOA4 through the ubiquitin-proteasome system. HMOX1 also contributes to the labile iron pool by catalyzing heme degradation. Iron is utilized in mitochondria for heme synthesis via FECH and ABCB6 or transported to the nucleus by PIR, which suppresses autophagy by limiting the cytosolic translocation of HMGB1. Excess iron can lead to oxidative stress and ROS production via the Fenton reaction, which can be countered by NQO1, HMOX1, or G6PD. ACSL4 is a key regulator of ferroptosis, producing PUFAs for subsequent ALOX-mediated lipid peroxidation. MGST1 inhibits ALOX activity through direct interaction, while MUFAs may competitively inhibit PUFA lipid peroxidation. Multiple mechanisms inhibit excessive lipid peroxidation, with GPX4 playing a central role by using GSH to neutralize lipid peroxides. GSH levels are positively regulated by the system xc^-^-mediated cystine uptake and subsequent cysteine production, with SLC7A11 and SLC3A2 as key components. GCL (composed of GCLC and GCLM) is the rate-limiting enzyme in GSH synthesis, and GSSG can be reduced back to GSH by GSR. AIFM2 provides a GPX4-independent anti-ferroptotic pathway through various mechanisms, including the production of CoQ10 or vitamin K, or activation of ESCRT-III membrane repair pathways. 4-HNE, an end product of lipid peroxidation, is detoxified by the ALDH family. NFE2L2-dependent genes are highlighted in green, while positive regulators of ferroptosis are shown in red. NFE2L2: NFE2-like basic leucine zipper transcription factor 2; TFRC: transferrin receptor; SLC40A1: solute carrier family 40 member 1; FTH1: ferritin heavy chain 1; FTL: ferritin light chain; MT1G: metallothionein 1G; NCOA4: nuclear receptor coactivator 4; HERC2: HECT and RLD domain containing E3 ubiquitin protein ligase 2; HMOX1: heme oxygenase 1; FECH: ferrochelatase; ABCB6: ATP-binding cassette subfamily B member 6; PIR: pirin; HMGB1: high mobility group box 1; ROS: reactive oxygen species; NQO1: quinone oxidoreductase-1; G6PD: glucose-6-phosphate dehydrogenase; ACSL4: acyl-CoA synthetase long-chain family member 4; PUFAs: polyunsaturated fatty acids; ALOX: lipoxygenase; MGST1: microsomal glutathione S-transferase 1; MUFAs: monounsaturated fatty acids; GPX4: glutathione peroxidase 4; GSH: glutathione; GCL: glutamate-cysteine ligase; GCLC: glutamate-cysteine ligase catalytic subunit; CGLM: glutamate-cysteine ligase modifier subunit; GSSG: oxidized glutathione; GSR: glutathione reductase; AIFM2: apoptosis-inducing factor mitochondria-associated 2; CoQ10: coenzyme Q10; ESCRT-III: endosomal sorting complex required for transport-III; 4-HNE: 4-hydroxynonenal; ALDH: aldehyde dehydrogenase.

The mechanisms underlying the induction and regulation of ferroptosis have been extensively studied. Despite its plasticity and heterogeneity, ferroptosis can be induced through two major pathways: the extrinsic (transporter-dependent) pathway and the intrinsic (enzyme-regulated) pathway^[24]^ [Figure 2]. For example, increased transferrin receptor (TFRC)-dependent iron uptake or inhibition of the system xc^-^-cystine/glutamate antiporter, leading to reduced GSH production, can trigger ferroptosis^[9]^. Additionally, the autophagic degradation of the iron storage protein ferritin, a process known as ferritinophagy, increases the labile iron pool and reactive oxygen species (ROS) production, thereby sensitizing cells to ferroptosis^[25,26]^.

The most studied intrinsic pathway involves GPX4, an enzyme that reduces lipid hydroperoxides to their corresponding alcohols using GSH as a substrate^[27]^. GPX4 activity is regulated by selenium^[28]^, and targeting GPX4 through covalent inhibitors, proteolysis-targeting chimeras, or cell-type-specific degraders can induce ferroptosis to suppress tumor growth^[29]^. Moreover, multiple GPX4-independent antioxidant enzymes, such as apoptosis-inducing factor mitochondria-associated 2 (AIFM2, also known as FSP1)^[30,31]^ and dihydroorotate dehydrogenase (DHODH)^[32]^, inhibit ferroptosis in cancer cells with low GPX4 expression. However, the role of AIFM2 or DHODH inhibitors in inducing ferroptosis through coenzyme Q10 (CoQ10) interference remains debated^[33]^. In certain contexts, AIFM2 inhibits ferroptosis by promoting the endosomal sorting complex required for transport-III (ESCRT-III)-mediated plasma membrane repair^[34]^ or by contributing to vitamin K production^[35-37]^, rather than solely affecting CoQ10.

The final stage of ferroptotic cell death is characterized by membrane rupture, although the precise mechanisms remain under investigation, including the possible involvement of pore-forming proteins^[38]^. It is generally accepted that excessive lipid peroxidation of polyunsaturated fatty acids (PUFAs), key components of cellular membranes, may directly lead to membrane rupture. The accumulation of toxic lipid metabolites may trigger a positive feedback loop, amplifying ferroptotic cell death and potentially spreading the damage to neighboring cells^[39]^.

Most ferroptosis inhibitors, such as ferrostatin-1 and liproxstatin-1, act by inhibiting the production of lipid ROS, thereby preventing lipid peroxidation. Acyl-CoA synthetase long-chain family member 4 (ACSL4) is a critical enzyme in the regulation of ferroptosis, as it facilitates the synthesis of PUFA-CoA esters^[40-43]^, which are highly susceptible to peroxidation by enzymes of the lipoxygenase (ALOX) family or cytochrome P450 oxidoreductase (POR)^[44,45]^. ACSL4 mediates at least two downstream pathways, leading to the formation of different PUFA-CoA esters. One pathway involves lysophosphatidylcholine acyltransferase 3 (LPCAT3), which incorporates PUFAs into phosphatidylethanolamines (PEs), making them prime targets for peroxidation^[40,43]^. The other pathway involves sterol O-acyltransferase 1 (SOAT1, also known as ACAT1), which produces PUFA-cholesteryl esters (CEs) instead of PUFA-PEs^[46]^. Both pathways contribute to lipid peroxidation, with the specific substrates used varying based on the cellular context.

In human pancreatic cancer cells lacking the lipid flippase solute carrier family 47 member 1 (SLC47A1), ACSL4-driven PUFA-CE production is particularly significant in promoting ferroptosis^[46]^. Conversely, elevated levels of monounsaturated fatty acids (MUFAs) inhibit ferroptosis, while saturated fatty acids may exert dual roles depending on the cellular environment^[47,48]^. For instance, the upregulation of LPCAT1 leads to the incorporation of more saturated fatty acids into membrane phospholipids, increasing phospholipid saturation and reducing PUFA content, thereby inhibiting ferroptosis^[49]^.

Lipid droplets - organelles that store neutral lipids such as triglycerides and cholesteryl esters - serve as a protective mechanism against ferroptosis by sequestering PUFAs or releasing MUFAs^[50]^. However, their degradation through lipophagy promotes ferroptosis by releasing PUFAs back into the cellular environment, where they become susceptible to peroxidation^[51]^. This organelle-level regulation of lipid metabolism presents a therapeutic target for modulating ferroptosis sensitivity in cancer cells. Given that lipid droplets contain both PUFAs and MUFAs, further investigation is needed to understand the signaling and mechanisms underlying the selective release of these fatty acids during ferroptosis.

Overall, the interaction between iron and lipid metabolism plays a complex role in regulating ferroptosis sensitivity. Understanding and modulating this pathway holds significant promise for developing new therapies for various diseases, particularly cancer.

## INVOLVEMENT OF AUTOPHAGY IN NFE2L2 ACTIVATION OR MODULATION

Autophagy is a lysosome-dependent degradation system that is essential for maintaining cellular homeostasis, particularly under stress conditions^[52,53]^. It facilitates the clearance of specific proteins and organelles, thereby playing a crucial role in determining cellular survival or death^[54,55]^. Generally, increased non-selective autophagy promotes cell survival by degrading damaged cellular components and recycling nutrients under stress conditions. However, selective autophagy, which targets specific cellular components, can either promote or inhibit cell death depending on the nature of the cargo being degraded. For instance, selective autophagy processes, such as ferritinophagy and lipophagy, promote ferroptosis by modulating iron and lipid metabolism, respectively^[25,51]^. In addition to these mechanisms, autophagy also plays a critical role in regulating NFE2L2 activation during ferroptosis through sequestosome 1 (SQSTM1, best known as p62) as first reported in HCC cells following treatment with erastin or sorafenib^[6]^.

SQSTM1 is a selective autophagy receptor that binds to ubiquitinated proteins and organelles, facilitating their degradation via the autophagy-lysosome pathway. SQSTM1 contains a ubiquitin-associated (UBA) domain that binds polyubiquitinated proteins, tagging them for autophagic degradation. Additionally, SQSTM1 interacts with microtubule-associated protein 1 light chain 3 (MAP1LC3, also known as LC3) through its LC3-interacting region (LIR). This interaction is critical for the sequestration of ubiquitinated cargo into autophagosomes, which are subsequently degraded upon fusion with lysosomes. For example, in HCC and HEK293T cells, SQSTM1 acts as a scaffold to promote the interaction between nuclear receptor coactivator 4 (NCOA4) and MAP1LC3, thereby facilitating ferritin degradation and enhancing sensitivity to ferroptosis^[56]^. This process is negatively regulated by the deubiquitinating enzyme ubiquitin-specific peptidase 8 (USP8)^[56]^.

**Figure 3:**
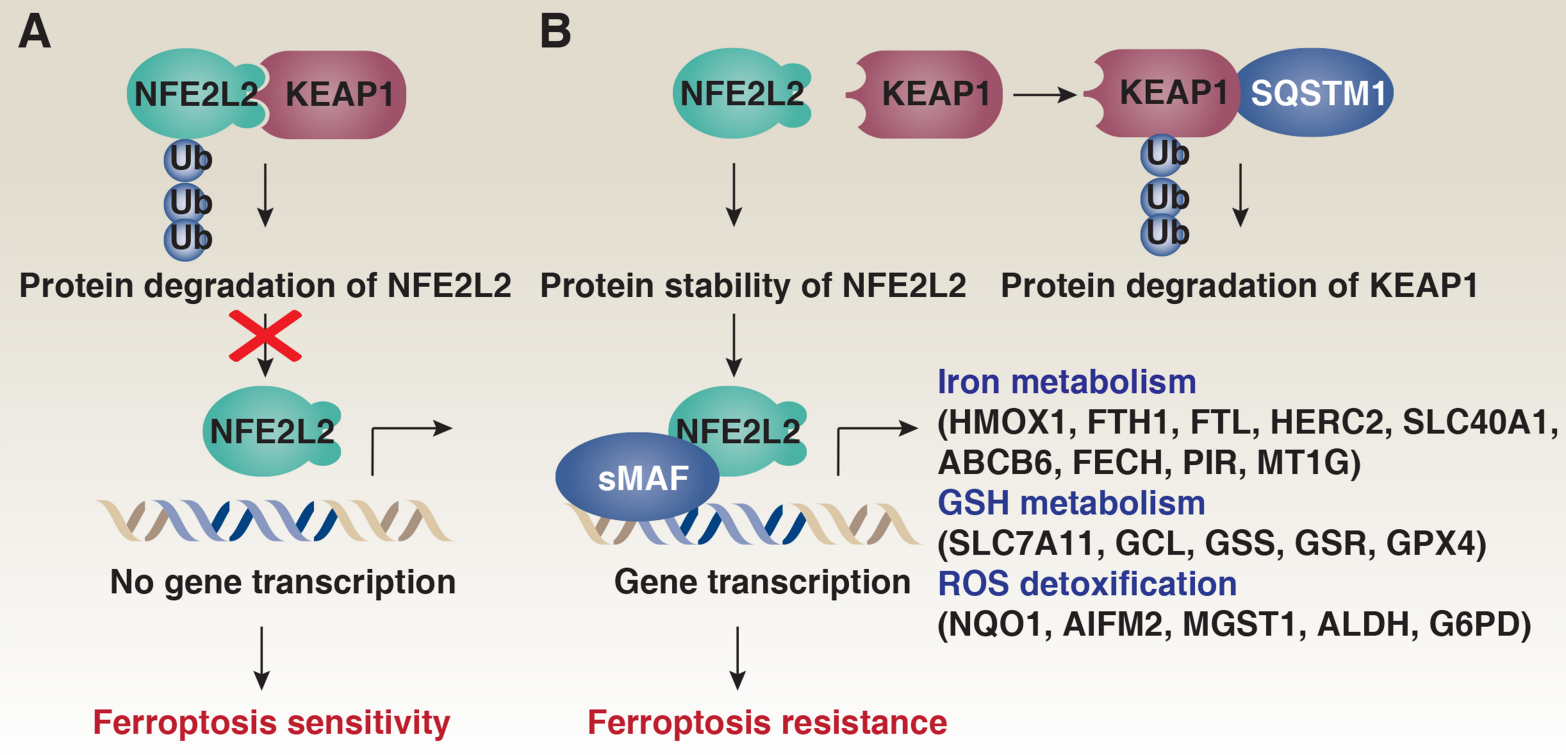
Effects of autophagy impairment on NFE2L2 activation in ferroptosis. (A) Under physiological conditions, NFE2L2 is continuously ubiquitinated by the KEAP1-CUL3 E3 ubiquitin ligase complex, resulting in its proteasomal degradation; (B) When autophagy is impaired, accumulated SQSTM1 interacts with KEAP1, leading to the proteasomal degradation of KEAP1. This interaction sequesters KEAP1 away from NFE2L2, preventing the ubiquitination and degradation of NFE2L2. Consequently, NFE2L2 is stabilized and translocates to the nucleus, where it dimerizes with sMAF proteins. This complex binds to AREs to mediate the transcription of genes involved in iron metabolism, GSH metabolism, and ROS detoxification. NFE2L2: NFE2-like basic leucine zipper transcription factor 2; KEAP1: Kelch-like ECH-associated protein 1; CUL3: cullin 3; SQSTM1: sequestosome 1; sMAF: small musculoaponeurotic fibrosarcoma; AREs: antioxidant response elements; GSH: glutathione; ROS: reactive oxygen species.

Autophagy deficiency leads to the accumulation of SQSTM1, which plays a role in positively regulating the transcription factor NFE2L2. Under normal conditions, NFE2L2 is continuously ubiquitinated by the KEAP1-CUL3-ring-box 1 (RBX1) E3 ubiquitin ligase complex, leading to its proteasomal degradation [Figure 3A]. However, when autophagy is impaired, accumulated SQSTM1 interacts with KEAP1 via its KEAP1-interacting region, effectively sequestering KEAP1 away from NFE2L2^[57]^ [Figure 3B]. This sequestration prevents the ubiquitination and degradation of NFE2L2, leading to its stabilization^[57]^. Indeed, RNAi-mediated knockdown of *SQSTM1* increases KEAP1 protein levels, enhances the interaction between KEAP1 and NFE2L2, and promotes the degradation of NFE2L2 in HCC cells in response to ferroptosis inducers, such as erastin and sorafenib^[6]^. Furthermore, the knockdown of *KEAP1* reverses the enhanced degradation of NFE2L2 observed following *SQSTM1* depletion in HCC cells after erastin treatment^[6]^. These findings demonstrate the critical role of the interaction between SQSTM1 and KEAP1 in maintaining NFE2L2 expression levels during ferroptosis.

Moreover, SQSTM1 undergoes phosphorylation at Ser349 in humans (Ser351 in mice), which enhances its binding affinity for KEAP1, further promoting the sequestration of KEAP1 and resulting in the activation of NFE2L2^[58]^. The activation of NFE2L2 under these conditions upregulates the expression of antioxidant response genes, protecting cells from oxidative stress and mitigating ferroptotic damage across various disease models^[6,59-62]^. As a feedback mechanism, the release of SQSTM1 during ferroptosis can increase advanced glycosylation end-product specific receptor (AGER, also known as RAGE)-dependent ACSL4 expression, leading to PUFA production for autophagosome formation and subsequent ferroptosis, as observed in pancreatitis models^[63]^.

In addition to the well-characterized KEAP1-CUL3-RBX1 complex, other E3 ligase complexes, such as the beta-transducin repeat containing E3 ubiquitin protein ligase (BTRC, also knowns as β-TrCP1)-S-phase kinase-associated protein 1 (SKP1)-CUL1-RBX1 complex and synoviolin 1 (SYVN1, also known as HRD1), are identified as mediators of NFE2L2 degradation during oxidative stress^[64,65]^. Although SYVN1 mediates erastin-induced ferroptosis in ovarian cancer cells by facilitating ubiquitination-dependent degradation of SLC7A11^[66]^, direct evidence for SYVN1-dependent NFE2L2 degradation during ferroptosis remains limited. It is essential to further investigate the specific signals that selectively activate these different E3 ligase complexes in the context of ferroptosis.

## NFE2L2-TARGETED GENES IN FERROPTOSIS AND DRUG RESISTANCE

The activation of NFE2L2 is pivotal in protecting cells from ferroptotic death. To achieve this protective function, NFE2L2 regulates a broad spectrum of genes involved in multiple cellular pathways, including those that modulate ROS, detoxify harmful agents, and repair damaged proteins [Figure 2 and Table 1]. Below, we summarize key NFE2L2-targeted genes that regulate ferroptosis inducer-mediated anticancer activity in a context-dependent manner.

**Table 1:** NFE2L2 target genes involved in the regulation of ferroptosis in cancer cells

**Name**	**General function**	**Role in ferroptosis**	**Tumor type**	**Ref.**
NQO1	Detoxification of quinones	Anti-ferroptotic by reducing ROS and protecting against lipid peroxidation	HCC, lung, pancreatic, and other cancers	[6]
HMOX1	Degradation of heme to biliverdin, iron, and CO	Dual role: promotes ferroptosis through free iron release, but can protect through antioxidant effects	Multiple cancers	[6,74-76]
FTH1	Iron storage	Anti-ferroptotic by sequestering free iron and preventing ROS formation	Multiple cancers	[6]
FTL	Iron storage	Works with FTH1 to prevent ferroptosis by reducing free iron levels	Multiple cancers	[6]
HERC2	E3 ubiquitin ligase regulating NCOA4 degradation	Anti-ferroptotic by inhibiting ferritinophagy, reducing iron availability	HCC	[81]
SLC40A1	Iron exporter	Anti-ferroptotic by reducing intracellular iron levels	Breast, neuroblastoma	[82,83]
ABCB6	Transport of porphyrins and detoxification	Inhibits ferroptosis by protecting cells from oxidative damage	Multiple myeloma	[88]
FECH	Heme synthesis	Inhibition leads to ferroptosis through reduced heme availability	Lung cancer	[89]
PIR	Oxidative stress response	Anti-ferroptotic by reducing DNA damage and inhibiting HMGB1 release	Pancreatic cancer	[92]
MT1G	Metal ion homeostasis and detoxification	Anti-ferroptotic by preventing lipid peroxidation	HCC, renal cell carcinoma	[94]
SLC7A11	Cystine/glutamate antiporter	Anti-ferroptotic by promoting GSH synthesis and ROS neutralization	Ovarian, esophageal, lung	[100,102,103]
GCL	Glutamate-cysteine ligase, GSH synthesis	Anti-ferroptotic by increasing GSH levels	Multiple cancers	[107]
GSS	GSH synthetase, GSH synthesis	Anti-ferroptotic by increasing GSH synthesis	Multiple cancers	[107]
GSR	GSH reductase, GSH regeneration	Anti-ferroptotic by regenerating reduced GSH	Multiple cancers	[107]
GPX4	Reduces lipid peroxides	Anti-ferroptotic by preventing lipid peroxidation	Multiple cancers	[113]
AIFM2	NAD(P)H oxidoreductase	Anti-ferroptotic by reducing CoQ10 and maintaining plasma membrane integrity	Lung, pancreatic, HCC	[30,31]
MGST1	Microsomal GSH S-transferase	Anti-ferroptotic by detoxifying lipid peroxidation products	Pancreatic, gastric, uterine	[116]
ALDH1A1	Detoxification of aldehydes	Anti-ferroptotic by detoxifying lipid peroxidation products	Breast, lung, cervical	[123]
ALDH3A1	Detoxification of aldehydes	Anti-ferroptotic by detoxifying lipid peroxidation products	Glioblastoma, colorectal	[123]
G6PD	NADPH production in the pentose phosphate pathway	Anti-ferroptotic by providing NADPH for GSH regeneration	Renal cell carcinoma, HCC	[129]

NFE2L2: NFE2-like basic leucine zipper transcription factor 2; NQO1: quinone oxidoreductase-1; ROS: reactive oxygen species; HCC: hepatocellular carcinoma; HMOX1: heme oxygenase 1; FTH1: ferritin heavy chain 1; FTL: ferritin light chain; HERC2: HECT and RLD domain containing E3 ubiquitin protein ligase 2; NCOA4: nuclear receptor coactivator 4; SLC40A1: solute carrier family 40 member 1; ABCB6: ATP binding cassette subfamily B member 6; FECH: ferrochelatase; PIR: pirin; HMGB1: high mobility group box 1; MT1G: metallothionein 1G; SLC7A11: solute carrier family 7 member 11; GSH: glutathione; GCL: glutamate-cysteine ligase; GSS: glutathione synthetase; GSR: glutathione reductase; GPX4: glutathione peroxidase 4; AIFM2: apoptosis-inducing factor mitochondria-associated 2; CoQ10: coenzyme Q10; MGST1: microsomal GSH S-transferase 1; G6PD: glucose-6-phosphate dehydrogenase; NADPH: nicotinamide adenine dinucleotide phosphate.

### Quinone oxidoreductase-1

Quinone oxidoreductase-1 (NQO1) is an enzyme in cellular defense against oxidative stress, primarily functioning within the body’s detoxification system. NQO1 catalyzes the two-electron reduction of quinones to hydroquinones, a process that mitigates the formation of ROS and detoxifies quinone-based drugs or those metabolized into quinone structures. NQO1 is frequently upregulated in various cancers, where its overexpression contributes to the development of drug resistance^[67]^. Elevated NQO1 levels in tumors correlate with poor prognosis, as the enzyme can reduce the efficacy of chemotherapy. Moreover, NQO1 upregulation has been proposed as a marker of aggressive tumor behavior.

In the context of ferroptosis, NFE2L2-mediated upregulation of NQO1 is implicated in promoting resistance to ferroptosis inducers, such as erastin and sorafenib, in HCC cells^[6]^. Conversely, genetic knockdown of *NQO1* enhances the sensitivity of HCC cells to these agents, underscoring its role in modulating ferroptosis susceptibility^[6]^. Inhibitors targeting NQO1 are being explored as potential therapeutic strategies to counteract its protective effects in cancer cells, thereby increasing their vulnerability to ferroptosis inducers.

Further complexity in NQO1’s role is highlighted by its interaction with various compounds. For instance, tanshinone acts as a coenzyme for NQO1, accepting electrons from flavin adenine dinucleotide to generate reduced tanshinone at the expense of nicotinamide adenine dinucleotide. This reduced form detoxifies lipid peroxyl radicals, thereby inhibiting erastin- or RSL3-induced ferroptosis in HT1080 cells^[68]^. In contrast, NQO1 is a predictive target for plumbagin using the PharmMapper database and molecular docking studies^[69]^. The growth-inhibitory effects of plumbagin are modulated by NQO1; suppression (via dicumarol) or elimination (via *NQO1* knockdown) of NQO1 reduces plumbagin-induced ferroptosis in glioma cells^[69]^. This reduction is attributed to plumbagin enhancing the interaction between NQO1 and tumor protein P53 (TP53, also known as p53), protecting TP53 from degradation and consequently reducing the expression of SLC7A11^[69]^. This pro-ferroptotic mechanism of NQO1 is independent of its antioxidant function. Moreover, the deletion of *NQO1* does not affect sensitivity to RSL3 alone^[31]^. However, cells lacking both *AIFM2* and *NQO1* exhibit increased sensitivity compared to *AIFM2* knockout U-2 OS cancer cells^[31]^.

Thus, NQO1 regulates ferroptosis sensitivity through multiple mechanisms involving both its enzymatic and non-enzymatic functions.

### Heme oxygenase 1

Heme oxygenase 1 (HMOX1, also known as HO-1), an NFE2L2-dependent inducible enzyme, plays a crucial role in the degradation of heme, a pro-oxidant, into three biologically active products: biliverdin, free iron, and carbon monoxide^[70]^. Each of these products has distinct biological activities that contribute to cellular protection. Biliverdin is rapidly converted to bilirubin, a potent antioxidant that scavenges ROS, thereby protecting cells from oxidative damage. Free iron released from heme is sequestered by ferritin, reducing the availability of free iron that could otherwise catalyze the formation of highly reactive hydroxyl radicals via the Fenton reaction. Carbon monoxide, with its anti-inflammatory and vasodilatory properties, further contributes to cellular protection against oxidative stress.

Through its antioxidant functions, particularly via bilirubin and carbon monoxide, HMOX1 mitigates oxidative stress and provides a protective advantage to cancer cells, shielding them from ferroptosis. For instance, in HCC cells, the knockdown of *HMOX1* via shRNA enhances the anticancer efficacy of erastin and sorafenib^[6]^. Preclinical studies have shown that inhibition of HMOX1 by zinc protoporphyrin or tin protoporphyrin enhances the efficacy of chemotherapy (e.g., cisplatin)^[71]^, photodynamic therapy^[72]^, and radiotherapy^[73]^. By inhibiting HMOX1, the protective effects on tumor cells are diminished, increasing their susceptibility to treatment-induced oxidative stress and subsequent cell death.

In some contexts, the free iron released by HMOX1 can enhance sensitivity to ferroptosis. For example, HMOX1 accelerates ferroptotic cell death induced by erastin, BAY 11-7085 (an IκBα inhibitor), or withaferin A in fibrosarcoma, breast cancer, and neuroblastoma cells, respectively^[74-76]^. Additionally, EF24, a synthetic analog of curcumin developed as an antitumor compound, induces ferroptosis in osteosarcoma cells by upregulating HMOX1, which in turn inhibits GPX4^[77]^. Co-treatment with donafenib and GSK-J4 also induces ferroptosis in HCC cells through the upregulation of HMOX1 expression^[78]^.

The dual role of HMOX1 in ferroptosis underscores the need for careful examination of the production of biliverdin, free iron, and carbon monoxide, as well as the buffering capacity of ferritin on free iron. Understanding these dynamics is essential for leveraging HMOX1 as a therapeutic target in cancer treatment.

### Ferritin heavy chain 1, ferritin light chain, HECT and RLD domain containing E3 ubiquitin protein ligase 2

Ferritin is a spherical protein complex composed of 24 subunits, which include two types: the heavy chain (FTH1) and the light chain (FTL). These subunits function cooperatively to store iron in a non-toxic, soluble form, thereby preventing the catalysis of harmful free radicals by free iron. Ferritin is thus crucial for maintaining iron homeostasis and protecting cells from iron-induced ferroptosis.

FTH1 was first identified as a target gene of NFE2L2 when it was observed that basal *Fth1* mRNA levels in *Nfe2l2*-deficient mice were significantly lower than those in wild-type mice^[79]^. The relationship between NFE2L2 activation and FTL was further elucidated when *Ftl* transcript levels were found to be twofold higher in WT compared to *Nfe2l2*-deficient mouse intestines^[80]^. In HCC cells, NFE2L2-mediated upregulation of *FTH1* enhances the cellular antioxidant defense by increasing the capacity for iron storage, thereby reducing erastin- or sorafenib-induced ferroptosis^[6]^. Conversely, ferritinophagy-mediated degradation of ferritin through autophagy receptor NCOA4 increases ferroptosis sensitivity, which suppresses the growth of various tumor cell types^[25]^. The NFE2L2-targeted gene HECT and RLD domain containing E3 ubiquitin protein ligase 2 (*HERC2*) encodes an E3 ubiquitin ligase that facilitates the proteasomal degradation of NCOA4, thereby inhibiting ferritinophagy-dependent ferroptosis^[81]^. Further insights into the regulatory mechanisms controlling ferritin induction and degradation may lead to the development of new ferroptosis-based antitumor strategies.

### Solute carrier family 40 member 1

Solute carrier family 40 member 1 (SLC40A1), also known as ferroportin-1, is a key iron-exporting protein responsible for transporting iron from the intracellular environment to the extracellular space. It is predominantly expressed in cells involved in iron storage and transport, including enterocytes, macrophages, hepatocytes, and placental cells. This iron-export function is critical for maintaining proper iron homeostasis across various tissues and within the bloodstream.

*SLC40A1* is a target gene of NFE2L2, with a putative ARE identified approximately -7 kb upstream of the *SLC40A1* core promoter. NFE2L2-mediated upregulation of SLC40A1 is anticipated to reduce intracellular iron levels and inhibit ferroptosis. For instance, overexpression of *SLC40A1* inhibits, while knockdown of *SLC40A1* enhances, siramesine and lapatinib-induced ferroptosis by modulating iron efflux in breast cancer cells^[82]^. Knockdown of *SLC40A1* also accelerates erastin-induced ferroptosis in neuroblastoma cells^[83]^, highlighting its role in modulating ferroptotic sensitivity. However, contradictory evidence exists; for instance, other studies have suggested that NFE2L2 induces cisplatin resistance by suppressing the iron export gene *SLC40A1* in ovarian cancer cells^[84]^, indicating the presence of unknown feedback mechanisms. Nonetheless, the degradation of SLC40A1 through SQSTM1-dependent autophagy or the ring finger protein 217 (RNF217)-dependent the ubiquitin-proteasome system enhances sensitivity to erastin- or RSL3-induced ferroptosis in certain cell types, including macrophages and HT1080 cells^[85,86]^.

In addition, the relationship between hepcidin and SLC40A1 forms a crucial feedback loop that maintains systemic iron homeostasis and modulates ferroptosis sensitivity^[83]^. Hepcidin negatively regulates SLC40A1 by promoting its degradation, thereby reducing iron export and controlling circulating iron levels. This balance is essential for preventing both iron deficiency and iron overload.

### ABCB6 and FECH ATP binding cassette subfamily B member 6, and ferrochelatase

Heme is a vital molecule consisting of a porphyrin ring complexed with ferrous iron, essential for various biological processes such as oxygen transport and electron transfer. Its synthesis occurs through a complex biochemical pathway involving multiple enzymatic steps and substrates. Disruptions or deficiencies in any of the enzymes or substrates required for heme synthesis can lead to the accumulation of intermediate compounds, resulting in a group of disorders known as porphyrias^[87]^. These disorders are characterized by the buildup of these intermediates in the blood, tissues, and urine, causing a range of clinical symptoms.

Two critical enzymes in this pathway, ATP binding cassette subfamily B member 6 (ABCB6) and ferrochelatase (FECH), are regulated by the transcription factor NFE2L2 and play significant roles in inhibiting ferroptosis when upregulated. ABCB6 acts as a broad-spectrum transporter of porphyrins, enabling the export and import of heme and its precursors across plasma and mitochondrial membranes. It also functions as a detoxifying agent by acting as a heavy metal efflux pump. Recent studies have identified nitidine chloride, a compound discovered through the screening of a natural product library, as a direct inhibitor of ABCB6^[88]^. This inhibition promotes ferroptosis and suppresses tumor growth in multiple myeloma by blocking the PI3K-AKT signaling pathway^[88]^.

FECH, another key enzyme in the heme biosynthesis pathway, catalyzes the insertion of ferrous iron into protoporphyrin IX to form heme B, a crucial step occurring within the matrix-facing side of the inner mitochondrial membrane. Interestingly, the flavonoid chalcone 4,4’-dimethoxychalcone induces ferroptosis in lung cancer cells by upregulating HMOX1 and inhibiting FECH^[89]^. This finding suggests that additional transcription factors may work alongside NFE2L2 to regulate the expression and activity of these enzymes, highlighting the complex regulation of heme synthesis and its impact on cell survival and death mechanisms.

### Pirin

Pirin (PIR) is a nuclear protein of the cupin superfamily, defined by a conserved β-barrel fold. It plays a crucial role in transcription regulation and oxidative stress response. As a metalloprotein with a non-heme iron binding site, PIR relies on this iron, which alternates between Fe^2+^ and Fe^3+^, to maintain its structural integrity and participate in redox reactions^[90]^.

In human pancreatic cancer cell lines, the expression of PIR is upregulated by NFE2L2 in response to ferroptosis-inducing agents (erastin or RSL3)^[91]^. Silencing PIR through knockdown experiments increases the levels of γ-H2AX, a marker of DNA damage, and 8-oxo-7,8-dihydro-2’-deoxyguanosine, an indicator of oxidative DNA damage, following treatment with these agents. This increased DNA damage is linked to increased cytoplasmic translocation and extracellular release of high mobility group box 1 (HMGB1) in *PIR*-deficient cancer cells^[91]^. Given that cytosolic HMGB1 can interact with Beclin 1 to activate autophagy-dependent ferroptosis^[92]^, the upregulation of PIR by NFE2L2 likely serves to retain HMGB1 in the nucleus, thereby inhibiting ferroptosis^[91]^.

Additionally, cancer cells with a pronounced epithelial-mesenchymal transition (EMT) phenotype - a process where polarized epithelial cells acquire mesenchymal characteristics - are more susceptible to ferroptosis^[93]^. Given PIR’s role in promoting EMT, investigating whether PIR-mediated EMT influences ferroptosis could offer valuable insights into the interplay between EMT and ferroptosis in cancer progression.

### Metallothionein 1G

Metallothionein 1G (MT1G) is a member of the metallothionein family, which comprises small, cysteine-rich proteins characterized by their ability to bind heavy metals through the thiol groups of cysteine residues. These metallothioneins play a critical role in maintaining metal ion homeostasis, protecting cells from metal toxicity, and facilitating the detoxification of harmful substances.

The expression of MT1G during ferroptosis is regulated by the NFE2L2 signaling pathway. In the context of cancer, particularly HCC, the upregulation of MT1G has been linked to resistance against sorafenib, a kinase inhibitor commonly used in cancer therapy^[94]^. The regulation of MT1G by sorafenib appears to be independent of its kinase inhibition activity. Instead, overexpression of *MT1G* reduces sorafenib-induced lipid peroxidation and subsequently inhibits ferroptosis. This protective effect of MT1G on cancer cells contributes to the development of drug resistance. Experimental evidence suggests that silencing or inhibiting MT1G expression can enhance the anticancer efficacy of sorafenib by promoting ferroptosis, both *in vitro* and *in vivo*^[94]^. This enhancement is particularly significant in clear cell renal cell carcinoma cell lines, such as 769-P and CAKI-1, where MT1G appears to modulate ferroptosis by regulating GSH consumption - a key antioxidant mechanism within these cells^[95]^.

Clinically, elevated levels of MT1G are associated with poor prognosis in patients undergoing treatment with sorafenib or immune checkpoint inhibitors for various cancers, including liver, colorectal, and prostate cancers^[96-98]^. These findings suggest that MT1G not only plays a role in mediating resistance to ferroptosis, but also serves as a potential biomarker for predicting patient outcomes and the effectiveness of ferroptosis-targeted therapies in cancer treatment. Consequently, targeting MT1G could represent a novel therapeutic strategy to overcome resistance and improve the efficacy of current anticancer treatments.

### SLC7A11

The heterodimeric amino acid transport system xc^-^ is composed of two subunits: the light chain subunit SLC7A11 and the heavy chain subunit SLC3A2. SLC7A11 specifically mediates the transport of extracellular cystine into the cell in exchange for intracellular glutamate, an essential function for maintaining the intracellular pool of cysteine. Cysteine, once imported, is rapidly reduced to cysteine, a key precursor in the synthesis of GSH. GSH is a critical intracellular antioxidant that protects cells from oxidative stress by neutralizing ROS and serves as a necessary cofactor for GPX4.

The regulation of SLC7A11 is intricately linked to the cellular response to oxidative stress and is tightly controlled by various signaling pathways, reflecting the context-dependent nature of its role in ferroptosis. TP53, a well-known tumor suppressor, negatively regulates SLC7A11, thereby sensitizing cells to ferroptosis by reducing cystine uptake and subsequent GSH synthesis^[99]^. This regulation by TP53 is particularly significant in the context of cancer, where the loss of TP53 function can lead to enhanced SLC7A11 activity, promoting tumor survival under oxidative stress conditions. Conversely, NFE2L2 and activating transcription factor 4 (ATF4) are positive regulators of SLC7A11^[100,101]^.

In esophageal squamous cell carcinoma, the upregulation of SLC7A11 by NFE2L2 is particularly prominent. NFE2L2 directly binds to the promoter region of SLC7A11, leading to increased expression of this transporter, which in turn contributes to the resistance to ferroptosis and to radiotherapy^[100]^. The therapeutic potential of targeting this pathway is highlighted by studies on brusatol, an inhibitor of NFE2L2. Brusatol induces ferroptosis in bladder cancer cells by suppressing NFE2L2-mediated upregulation of SLC7A11, thereby reducing the cell’s antioxidant capacity^[102]^. The expression of SLC7A11 is also upregulated in ovarian cancer tissues, playing a critical role in promoting resistance to ferroptosis by enhancing cystine uptake and supporting GSH biosynthesis^[103]^. Overall, modulating the NFE2L2-SLC7A11 axis could be a viable strategy for overcoming resistance to ferroptosis in certain cancers.

Moreover, the transcriptional regulation of *SLC7A11* is further influenced by NFE2L2-binding cofactors, such as alternate reading frame (ARF), which is a key mediator of TP53 activation in response to oncogenic signals^[104]^, and YEATS domain containing 4 (YEATS4, also known as GAS41), a member of the YEATS family of chromatin-associated proteins^[105]^. These cofactors enhance the transcriptional activity of NFE2L2, thereby amplifying the expression of SLC7A11 and contributing to the inhibition of ferroptosis in cancer cells^[104,105]^. This amplification of SLC7A11 expression through cofactor interaction underscores the complex regulatory network that governs the cellular response to oxidative stress and ferroptosis.

Phosphatase and tensin homolog deleted on chromosome 10 (PTEN), one of the most frequently mutated tumor suppressors in cancer, also plays a critical role in modulating SLC7A11 expression. The loss of *PTEN* function, commonly observed in various cancers, leads to increased expression of SLC7A11^[106]^. This occurs through the prevention of BTRC-mediated degradation of NFE2L2, which in turn sustains high levels of SLC7A11, thereby reducing the susceptibility of cancer cells to ferroptosis^[106]^. The elevated SLC7A11 expression associated with PTEN loss is thought to confer a survival advantage to tumor cells by enhancing their antioxidant defenses, thereby promoting tumorigenesis and resistance to therapy.

### glutamate-cysteine ligase, GSH synthetase, and GSH-disulfide reductase

The initial and rate-determining step in GSH synthesis is facilitated by glutamate-cysteine ligase (GCL, formerly termed γ-glutamylcysteine synthetase). GCL is a heterodimeric enzyme composed of two subunits: the catalytic subunit (GCLC) and the modifier subunit (GCLM), which are encoded by distinct genes. GCLC mediates the formation of a γ-carboxyl linkage between glutamate and cysteine, a reaction that necessitates ATP and Mg^++^ as cofactors. GCLM enhances the catalytic efficiency and turnover number of GCLC, lowers the Michaelis constant for glutamate and ATP, and increases the inhibition constant for GSH-mediated feedback inhibition of GCL. The subsequent enzyme, GSH synthetase (GSS), in the GSH biosynthetic pathway catalyzes the conjugation of gamma-glutamylcysteine with glycine to generate GSH. GSH-disulfide reductase (GSR) then catalyzes the reduction of oxidized GSH (GSSG) back to its reduced form (GSH), a pivotal molecule in counteracting oxidative stress and preserving the cell’s redox balance.

Importantly, GCLC, GCLM, GSS, and GSR are transcriptional targets of NFE2L2. Their upregulation is implicated in conferring resistance to ferroptosis across various contexts, including chemotherapy and radiation therapy^[107]^. While the GSH pathway and its involvement in ferroptosis have garnered increasing attention, GSH also serves as an inhibitor of other forms of regulated cell death, such as apoptosis and cuproptosis. Further elucidating the specific roles of GSH-dependent enzymes and identifying their targets are critical for differentiating the diverse roles of GSH in cell death processes.

### GPX4

GPX4 stands out within the GPX family due to its unique ability to reduce lipid hydroperoxides directly within cell membranes, thereby safeguarding cells from lipid peroxidation and ferroptosis. Elevated GPX4 expression is linked to resistance against chemotherapy, highlighting its role in drug resistance. Conversely, pharmacological inhibition of GPX4 can induce ferroptosis, offering a potential strategy to overcome therapy resistance. Impaired protein degradation pathways, including the ubiquitin-proteasome system and autophagy, significantly contribute to the upregulation of GPX4. Moreover, GPX4 expression is regulated at the transcriptional level, and it has been identified as a potential target gene of NFE2L2. Several enzymes involved in protecting against ROS, including GPX4, are upregulated in wild-type mice but not in *Nfe2l2* knockout mice treated with chemopreventive agents, such as 3H-1,2-dithiole-3-thione, isothiocyanate PEITC, and sulforaphane^[80,108,109]^.

Inhibition of NFE2L2 by trigonelline reverses resistance to GPX4 inhibitor-induced ferroptosis in head and neck cancer cells^[110]^, implicating NFE2L2 in the development of resistance to GPX4 inhibitors. This resistance is linked to the induction of sestrin 2 (SESN2) expression under endoplasmic reticulum stress and subsequent degradation of KEAP1 via SQSTM1, establishing a connection between endoplasmic reticulum stress, NFE2L2 activity, and ferroptosis resistance^[110]^. *SESN2* itself is an NFE2L2-dependent gene, with its expression induced by NFE2L2 activators such as tert-butylhydroquinone and sulforaphane in hepatocytes^[111]^, suggesting a positive feedback loop between SESN2 expression and NFE2L2 activation^[112]^.

In breast cancer cells, NFE2L2-mediated transcriptional activation of GPX4 contributes to radioresistance^[113]^. Interestingly, tubastatin A, a selective HDAC6 inhibitor, overcomes both ferroptosis resistance and radioresistance in cancer cells by inhibiting GPX4 enzymatic activity, independent of histone deacetylase 6 (HDAC6) inhibition^[113]^. Further research is needed to elucidate the precise mechanisms by which tubastatin A inhibits GPX4 activity and to compare its effects with known GPX4 inhibitors such as RSL3.

Overall, GPX4 is a key driver of ferroptosis resistance; however, its expression also influences other forms of non-ferroptotic cell death, such as pyroptosis. The molecular mechanisms underlying various cell death pathways induced by GPX4 deficiency require further investigation.

### AIFM2

AIFM2, a flavoprotein, functions as an NAD(P)H-dependent oxidoreductase with significant roles beyond its initial hypothesized function. Originally, it was believed that AIFM2 might induce apoptosis within mitochondria due to its structural similarity to apoptosis-inducing factor mitochondria-associated 1 (AIFM1). However, recent research has revealed that AIFM2 plays a critical role in a GPX4-independent ferroptosis defense mechanism, particularly in *GPX4*-knockout cells^[30,31]^. This defense mechanism is localized to the plasma membrane, where AIFM2 is myristoylated, facilitating its membrane localization and enabling it to function effectively as an oxidoreductase that reduces CoQ10.

CoQ10 is a fat-soluble molecule ubiquitous in human cells, where it is essential for energy production through its involvement in the mitochondrial electron transport chain. Moreover, CoQ10 serves as a potent antioxidant, neutralizing free radicals and thereby protecting cellular membranes from oxidative damage. This antioxidant function of CoQ10 is crucial in the context of ferroptosis. In addition to its role in CoQ10 reduction, increased expression of AIFM2 has been associated with the activation of the ESCRT-III repair machinery^[34]^. This activation is crucial for maintaining membrane integrity by mitigating oxidative damage. Furthermore, AIFM2 is linked to the production of reduced vitamin K, another important factor in protecting cellular membranes from oxidative stress^[35,37]^.

There is a positive correlation between AIFM2 expression and the sensitivity of cancer cells to ferroptosis inducers such as RSL3, ML162, ML210, and erastin. This relationship suggests that targeting AIFM2 could be a promising strategy to overcome ferroptosis resistance in cancer therapy, particularly in tumors that are resistant to conventional treatments. Upregulation of AIFM2 has also been implicated in the activation of the NFE2L2 pathway^[114]^. Two AREs are identified in the promoter regions of the *AIFM2* gene, indicating that AIFM2 expression is tightly regulated by NFE2L2. Targeting NFE2L2-dependent AIFM2 expression has shown potential in enhancing ferroptosis-mediated tumor suppression, especially in lung cancer cells deficient in *KEAP1*^[114]^.

However, further research is necessary to fully understand the context-dependent roles of NFE2L2-mediated AIFM2 expression under various ferroptotic stresses. For example, a recent study demonstrated that AIFM2 confers ferroptosis resistance in KEAP1-mutant non-small cell lung carcinoma through both NFE2L2-dependent and independent mechanisms^[115]^. Moreover, it would be beneficial to explore the mechanisms by which *GPX4* depletion triggers the AIFM2-mediated anti-ferroptotic pathway, contributing to cell survival. Understanding these pathways could provide new therapeutic targets for cancer treatment and potentially improve the efficacy of ferroptosis-based therapies.

### Microsomal GSH S-transferase 1

Microsomal GSH S-transferase 1 (MGST1) is a membrane-bound enzyme primarily localized in the mitochondria, endoplasmic reticulum, plasma membrane, and peroxisomes. MGST1 exhibits dual functions, acting both as a GSH S-transferase (GST) and as a GPX, enabling it to metabolize a wide range of substrates. These substrates include products of lipid peroxidation, halogenated hydrocarbons, and carcinogenic drugs, highlighting its role in cellular detoxification processes.

Beyond its detoxifying capabilities, MGST1 plays a critical role in inhibiting apoptosis. Inducible MGST1 expression, mediated by the activation of the NFE2L2 pathway, inhibits ferroptosis in pancreatic cancer cells^[116]^. However, mutations in the ARE of the *MGST1* gene can impair its upregulation during ferroptosis, weakening this protective effect. Functionally, the loss of MGST1, similar to the loss of NFE2L2, reduces the resistance of pancreatic cancer cells to ferroptosis, making them more vulnerable to this form of cell death. Conversely, re-expression of MGST1 in *NFE2L2*-knockdown pancreatic ductal adenocarcinoma cells can restore their resistance to ferroptosis, underscoring the critical role of MGST1 in ferroptosis regulation. Mechanistically, MGST1 has been shown to interact with arachidonate 5-lipoxygenase (ALOX5), inhibiting ALOX5-mediated lipid peroxidation, a key driver of ferroptosis^[116]^.

The implications of MGST1 extend beyond pancreatic cancer. Elevated MGST1 expression is associated with poor prognosis and ferroptosis resistance in other cancers, including gastric cancer and uterine corpus endometrial carcinoma^[117,118]^. In gastric cancer cells, *MGST1* overexpression leads to the inhibition of autophagy, a process that can trigger autophagy-dependent ferroptosis^[119]^. Specifically, MGST1 represses the expression of autophagy-related 16-like 1 (ATG16L1) and the conversion of MAP1LC3-I to MAP1LC3-II, both critical steps in autophagy, thereby providing an additional mechanism by which MGST1 confers ferroptosis resistance^[119]^.

In addition to NFE2L2, the transcription factor zinc finger protein 384 (ZNF384) has also been implicated in the upregulation of MGST1^[120]^. In glioma cells, ZNF384-mediated MGST1 expression contributes to resistance against the chemotherapeutic agent temozolomide by negatively regulating ferroptosis^[120]^. This highlights the potential of MGST1 as a target for overcoming chemoresistance in glioma.

Collectively, these studies suggest that targeting MGST1, either through NFE2L2-dependent or NFE2L2-independent pathways, represents a promising strategy to enhance ferroptosis-mediated tumor suppression. This approach could potentially improve the efficacy of cancer therapies, particularly in cancers that exhibit resistance to ferroptosis.

### ALDH1A1 and ALDH3A1

The aldehyde dehydrogenase 1 (ALDH1) family, a group of isoenzymes within the acetaldehyde dehydrogenase superfamily, contributes to cellular detoxification processes. These enzymes are primarily responsible for the oxidation of acetaldehyde, a toxic byproduct of alcohol metabolism, into acetic acid via alcohol dehydrogenase. This conversion is crucial for maintaining cellular homeostasis and preventing the accumulation of harmful aldehydes that can lead to cellular damage and disease.

Among the ALDH1 family members, ALDH1B1 has garnered particular interest due to its interactions with eukaryotic translation initiation factor 4E (EIF4E) within membrane compartments, especially mitochondria-associated membranes^[121]^. This interaction inhibits the ALDH1B1-mediated clearance of 4-hydroxynonenal (4-HNE), a lipid peroxidation byproduct and a potent mediator of ferroptosis. By limiting the detoxification of 4-HNE, the EIF4E-ALDH1B1 complex increases cellular susceptibility to ferroptosis^[121]^.

The ALDH1 family comprises six functional genes, each with distinct biological roles. Among these, ALDH1A1 and ALDH3A1 have been identified as direct targets of the NFE2L2 pathway. The upregulation of these genes is associated with drug resistance in various cancers. For example, *ALDH1A1* overexpression is linked to resistance against several chemotherapeutic agents, including cyclophosphamide, doxorubicin, and paclitaxel in breast cancer^[122]^; paclitaxel in lung cancer^[123]^; and cisplatin in cervical cancer^[124]^. This resistance mechanism is partly due to the ability of ALDH1A1 to detoxify aldehydes generated by chemotherapy, thereby protecting cancer cells from cytotoxicity. In colorectal cancer, long-term exposure to increasing concentrations of 5-fluorouracil induces a switch from ALDH1A1 to the ALDH1A3 isoform^[125]^. This isoform switch is associated with cross-resistance to multiple chemotherapeutic agents, including 5-fluorouracil, oxaliplatin, cisplatin, and irinotecan, making treatment more challenging and reducing the efficacy of conventional therapies^[125]^.

Therapeutically, targeting NFE2L2-dependent expression of ALDH1A1 and ALDH1A3 offers a promising strategy to enhance the sensitivity of cancer cells to chemotherapy. In pancreatic cancer cells, inhibition of these isoenzymes increases the antiproliferative effects of 5-fluorouracil^[126]^. Additionally, the inhibition of ALDH1A1 reduces the stemness of human and mouse glioblastoma cells by inducing ferroptosis, suggesting a potential strategy for targeting cancer stem cells and overcoming resistance^[127]^.

Interestingly, the microbial metabolite indole-3-acetaldehyde has been reported to upregulate ALDH3A1, which catalyzes the conversion of retinal into nicotinamide adenine dinucleotide^[128]^. This reaction is crucial for powering the AIFM2-mediated antiferroptosis system, which contributes to the development and progression of colorectal cancer^[128]^. Moreover, the upregulation of ALDH3A1 is implicated in the development of temozolomide-resistant glioblastoma cells, primarily due to impaired ferroptosis, further highlighting the role of ALDH3A1 in cancer resistance mechanisms.

Given the diverse roles of the ALDH1 family in mediating drug resistance, particularly through NFE2L2-dependent pathways, targeting these enzymes represents a potent therapeutic approach. By inhibiting ALDH1A1 and ALDH3A1, it may be possible to overcome resistance to chemotherapy and enhance the efficacy of existing cancer treatments, providing new avenues for combating resistant cancer types.

### Glucose-6-phosphate dehydrogenase

Glucose-6-phosphate dehydrogenase (G6PD) is an enzyme that catalyzes the rate-limiting first step of the pentose phosphate pathway (PPP), an essential metabolic route that operates parallel to glycolysis. The primary function of the PPP is to generate reducing equivalents in the form of nicotinamide adenine dinucleotide phosphate (NADPH) and to produce ribose-5-phosphate, a precursor for nucleotide synthesis. G6PD facilitates the conversion of glucose-6-phosphate into 6-phosphoglucono-δ-lactone, simultaneously reducing NADP^+^ to NADPH. This step is vital as NADPH is a crucial cofactor in various anabolic reactions and is indispensable for maintaining cellular redox balance.

NADPH plays a fundamental role in protecting cells against oxidative stress by serving as a reducing agent in the regeneration of GSH and thioredoxin, two critical antioxidants that neutralize ROS. The ability of NADPH to sustain antioxidant defenses is particularly important in red blood cells (RBCs), which are continuously exposed to high levels of oxygen and lack mitochondria, the primary source of alternative NADPH production. Without sufficient NADPH, RBCs are highly vulnerable to oxidative damage, which can result in hemolysis and various hemolytic disorders, particularly in individuals with G6PD deficiency^[129]^.

While *G6PD* deficiency is associated with hemolytic anemia, it has also been linked to a lower risk and mortality for certain types of cancer. This observation suggests a complex role of G6PD in cancer biology, where its activity may be crucial for the survival and proliferation of cancer cells. Many cancers exhibit upregulated G6PD expression, which enhances their ability to cope with oxidative stress, supports rapid cell growth, and contributes to metabolic reprogramming that favors tumor progression.

In cancer cells, the overexpression of G6PD and the subsequent activation of the pentose phosphate pathway are often driven by oncogenic signaling pathways, including the NFE2L2 pathway. The upregulation of G6PD through NFE2L2 activation not only aids in the detoxification of ROS but also contributes to the resistance of cancer cells to ferroptosis^[130]^. This resistance to ferroptosis is particularly evident in clear cell renal cell carcinoma and HCC, where elevated G6PD expression and PPP activation have been shown to protect against oxidative damage and promote cancer cell survival. Further understanding the regulation and function of G6PD in various cancers may offer new insights into therapeutic strategies aimed at targeting metabolic vulnerabilities and overcoming resistance to ferroptosis, potentially leading to more effective cancer treatments^[131]^.

## TARGETING NFE2L2-ASSOCIATED FERROPTOSIS REGULATION IN CANCER THERAPY

Integrating NFE2L2-associated ferroptosis regulation into cancer therapies is critical for advancing the clinical potential of this research in oncology. NFE2L2 plays a central role in promoting resistance to ferroptosis by upregulating key antioxidant defenses, such as GPX4 and SLC7A11, which neutralize ROS and lipid peroxides. This defense mechanism provides cancer cells with a survival advantage, especially in oxidative and stress-inducing environments, highlighting the therapeutic potential of targeting NFE2L2. Inhibition of NFE2L2 can sensitize cancer cells to ferroptosis-inducing agents, thereby enhancing the efficacy of chemotherapy, radiotherapy, and targeted therapies that rely on oxidative stress to eliminate tumor cells^[132-137]^. Furthermore, combining NFE2L2 inhibition with immunotherapies (e.g., CD19-targeted CAR T-cell therapeutics) could amplify tumor immunogenicity by increasing ROS accumulation and ferroptotic cell death, thereby boosting antitumor immune responses^[138]^. These strategies, when employed in conjunction with existing treatments such as radiotherapy and targeted drug delivery systems, offer the potential for more comprehensive and durable cancer therapies by addressing both tumor survival mechanisms and NFE2L2-driven resistance pathways^[139,140]^.

Despite the progress in understanding NFE2L2-regulated genes involved in ferroptosis, there remains a gap in the systematic investigation of each gene’s specific contribution to ferroptosis regulation. Among these genes, GPX4 is of particular interest due to its critical role in reducing lipid peroxides and promoting ferroptosis resistance. Targeting the GPX4 pathway holds significant therapeutic potential, particularly when combined with standard treatments such as chemotherapy and radiotherapy, which are less effective in apoptosis-resistant tumors. However, current GPX4 inhibitors face challenges such as poor metabolic stability, limited bioavailability, and side effects *in vivo*^[141,142]^. Recent advancements in pharmacological approaches to modulate GPX4 activity and expression, such as covalent inhibitors (e.g., LOC1886), proteolysis targeting chimera degraders (e.g., dGPX4), and cell-type-specific degraders (e.g., N6F11), offer promising strategies for inducing ferroptosis as a therapeutic intervention in cancer treatment^[29]^. These novel approaches offer the potential to overcome the limitations of existing inhibitors, enhancing both efficacy and safety in clinical applications. Expanding research in this area is critical for optimizing ferroptosis modulation and may pave the way for innovative therapeutic strategies that utilize NFE2L2 inhibition to overcome drug resistance and improve clinical outcomes across various cancer types.

## CONCLUSIONS AND PERSPECTIVES

Ferroptosis is an iron-related form of oxidative stress driven by excessive lipid peroxidation, and its mechanisms and regulation are highly context-dependent^[143]^. The activation of the SLC7A11-GSH-GPX4 pathway plays a central role in defending against ferroptosis. In cells with low GPX4 expression, GPX4-independent pathways, such as AIFM2, are more effective in inhibiting ferroptosis. However, conditions where both GPX4-dependent and independent systems are active also occur in cells or tissues with normal GPX4 expression. These phenotypes reinforce the notion that the body possesses multiple antioxidant systems that can function either dependently or independently to combat oxidative stress.

Nonetheless, overactivation of NFE2L2-regulated genes plays a critical role in both GPX4-dependent and independent antioxidant pathways, contributing to key processes such as scavenging ROS, synthesizing GSH, producing NADPH, metabolizing heme and iron, detoxifying xenobiotics, and chelating metals. Moreover, NFE2L2-driven metabolic reprogramming maintains redox homeostasis, further promoting resistance to therapy. Therefore, therapeutic strategies aimed at modulating NFE2L2 activity could offer more effective means of regulating ferroptosis and overcoming drug resistance than current approaches. A deeper understanding of the interplay between NFE2L2 signaling, ferroptosis regulation, and resistance mechanisms is essential for the development of novel cancer therapies.

Despite the exciting advances in understanding NFE2L2 and ferroptosis, several challenges and questions remain to be addressed^[142]^. First, there is a lack of comprehensive studies examining the dynamics of NFE2L2-target gene induction during ferroptosis. This is crucial because some target genes act as early-response regulators, while others function as late-response regulators. Second, based on current literature, the discussed NFE2L2-regulated genes also modulate other forms of oxidative cell death. It remains unclear how these target genes selectively regulate different cell death pathways. Third, excessive production of reduced antioxidants can also trigger cell death, a phenomenon we previously termed “reductive cell death”. It is necessary to evaluate the threshold at which antioxidants switch from anti-death to pro-death functions. Finally, the current NFE2L2 inhibitors and activators generally affect the overall function of NFE2L2. There is a need for collaboration between industry and academia to design next-generation NFE2L2-related compounds that can selectively modulate NFE2L2 expression or function in specific tissues or cells, or at the level of individual NFE2L2-targeted genes. This specificity is crucial for reducing side effects.

## DECLARATIONS

### Copyright

© The Author(s) 2024.
